# Matching disease and phenotype ontologies in the ontology alignment evaluation initiative

**DOI:** 10.1186/s13326-017-0162-9

**Published:** 2017-12-02

**Authors:** Ian Harrow, Ernesto Jiménez-Ruiz, Andrea Splendiani, Martin Romacker, Peter Woollard, Scott Markel, Yasmin Alam-Faruque, Martin Koch, James Malone, Arild Waaler

**Affiliations:** 1 Pistoia Alliance Ontologies Mapping Project, Pistoia Alliance Inc, USA; 2 Department of Informatics, University of Oslo, Oslo, Norway; 3 Novartis, Basel, Switzerland; 4 Roche Pharma Research and Early Development, pRED Informatics, Roche Innovation Center, Basel, Switzerland; 5 GlaxoSmithKline R&D, Stevenage, UK; 6 BIOVIA 3DS, San Diego, USA; 7 Eagle Genomics, Cambridge, UK; 8 OSTHUS, Aachen, Germany; 9 FactBio, Cambridge, UK

**Keywords:** Biomedical ontology, Ontology alignment, OAE, Evaluation, Phenotype, Disease

## Abstract

**Background:**

The disease and phenotype track was designed to evaluate the relative performance of ontology matching systems that generate mappings between source ontologies. Disease and phenotype ontologies are important for applications such as data mining, data integration and knowledge management to support translational science in drug discovery and understanding the genetics of disease.

**Results:**

Eleven systems (out of 21 OAEI participating systems) were able to cope with at least one of the tasks in the *Disease and Phenotype* track. AML, FCA-Map, LogMap(Bio) and PhenoMF systems produced the top results for ontology matching in comparison to consensus alignments. The results against manually curated mappings proved to be more difficult most likely because these mapping sets comprised mostly subsumption relationships rather than equivalence. Manual assessment of unique equivalence mappings showed that AML, LogMap(Bio) and PhenoMF systems have the highest precision results.

**Conclusions:**

Four systems gave the highest performance for matching disease and phenotype ontologies. These systems coped well with the detection of equivalence matches, but struggled to detect semantic similarity. This deserves more attention in the future development of ontology matching systems. The findings of this evaluation show that such systems could help to automate equivalence matching in the workflow of curators, who maintain ontology mapping services in numerous domains such as disease and phenotype.

## Background

The Pistoia Alliance Ontologies Mapping project^1^ was set up to find or create better tools and services for mapping between ontologies (including controlled vocabularies) in the same domain and to establish best practices for ontology management in the Life Sciences. The project has developed a formal process to define and submit a request for information (RFI) from ontology matching system providers to enable their evaluation.^2^ A critical component of any ontology alignment system is the embedded matching algorithm, therefore the Ontologies Mapping project is supporting their development and evaluation through sponsorship and organisation of the *Disease and Phenotype* track (added in 2016) for the OAEI campaign [1]. In this paper we describe the experiences and results in the OAEI 2016 *Disease and Phenotype* track.^3^

The *Disease and Phenotype* track is based on a real use case where it is required to find two pairwise alignments between disease and phenotype ontologies: *(i)* Human Phenotype Ontology [2] (HP) to Mammalian Phenotype Ontology [3] (MP), and *(ii)* Human Disease Ontology [4] (DOID) to Orphanet Rare Disease Ontology^4^ (ORDO). The first task maps between human and the more general mammalian phenotype ontologies. This is important for translational science in drug discovery, since mammalian models such as mice are widely used to study human diseases and their underlying genetics. Mapping human phenotypes to other mammalian phenotypes greatly facilitates the extrapolation from model animals to humans. The second task maps between two disease ontologies: the more generic DOID and the more specific ORDO, in the context of rare human diseases. These ontologies can support investigative studies to understand how genetic variation can cause or contribute to disease.

Currently, mappings between the aforementioned ontologies within the disease and phenotype domain are mostly generated manually by bioinformaticians and disease experts. Inclusion of automated ontology matching systems into such curation workflows is likely to improve the efficiency and scalability of this process to expand the coverage across many source ontologies. Automation of mappings is also important because the source ontologies are dynamic, often having more than ten versions per year which means the mappings must be maintained to remain useful and valid.

### Preliminaries

In this paper we assume that the ontologies are represented using the OWL 2 Web Ontology Language [5], which is a World Wide Web Consortium (W3C) recommendation.^5^ Description Logics (DL) are the formal underpinning of OWL 2 [6].

An ontology *mapping* (also called *match* or *correspondence*) between entities of two ontologies ${\mathcal {O}_{1}}, {\mathcal {O}_{2}}$ is typically represented as a 4-tuple 〈*e*,*e* ^′^,*r*,*c*〉 where *e* and *e* ^′^ are entities of ${\mathcal {O}_{1}}$ and ${\mathcal {O}_{2}}$, respectively; $r \in \{\sqsubseteq, \sqsupseteq, \equiv \}$ is a semantic relation; and *c* is a confidence value, usually, a real number within the interval (0…1]. Mapping confidence intuitively reflects how reliable a mapping is (i.e., 1 = very reliable, 0 = not reliable).

An ontology *alignment* $\mathcal {M}$ between two ontologies, namely $\mathcal {O}_{1}, \mathcal {O}_{2}$, is a set of mappings between $\mathcal {O}_{1}$ and $\mathcal {O}_{2}$. In the ontology matching community, mappings are typically expressed using the RDF Alignment format [7].

In addition, mappings can also be represented through standard OWL 2 axioms (e.g., [8]). This representation enables the use of the OWL 2 reasoning infrastructure that is currently available.

When mappings are translated into OWL 2 axioms, an *aligned ontology* ${\mathcal {O}^{\mathcal {M}}}=\mathcal {O}_{1} \cup \mathcal {O}_{2} \cup \mathcal {M}$ is the result of merging the input ontologies and an alignment between them. The aligned ontology is also an OWL 2 ontology.

An ontology *matching system* is a program^6^ that, given two input ontologies $\mathcal {O}_{1}$ and $\mathcal {O}_{2}$, generates an ontology alignment $\mathcal {M}^{S}$.

An ontology *matching task* is typically composed by one or more pairs of ontologies with their correspondent *reference alignments* $\mathcal {M}^{R\!A}$. Reference alignments can be of different nature: gold standards, silver standards and baselines. *Gold* *standards* are typically (almost) complete mapping sets that have been manually curated by domain experts, while *silver* *standard* mapping sets are not necessarily complete nor correct. Finally, *baseline* mappings typically represent a highly incomplete set of the total mappings. In this paper we use a type of *silver* *standard* that has been created by voting the mappings produced by several matching systems. In the remainder of the paper, we refer to this (silver standard) mapping set as *consensus* *alignments*.

The standard evaluation measures, for a system generated alignment $\mathcal {M}^{S}$, are *precision* (P), *recall* (R) and *f-measure* (F) computed against a reference alignment $\mathcal {M}^{RA}$ as follows:

1 $$ P = \frac{\lvert\mathcal{M}^{S} \cap \mathcal{M}^{RA}\rvert}{\lvert\mathcal{M}^{S}\rvert},~ R = \frac{\lvert\mathcal{M}^{S} \cap \mathcal{M}^{RA}\rvert}{\lvert\mathcal{M}^{RA}\rvert},~ F = 2 \cdot \frac{P \cdot R}{P + R}  $$

Standard precision and recall have, however, limitations when considering the (OWL 2) semantics of the input ontologies and the mappings. Hence a mapping *m* such that $m \in \mathcal {M}^{S}$ and $m \not \in \mathcal {M}^{RA}$ will penalise the standard precision value even though $\mathcal {O}^{\mathcal {M}^{RA}} \models m$, that is, *m* is inferred or entailed (using OWL 2 reasoning) by the union of the input ontologies $\mathcal {O}_{1}$ and $\mathcal {O}_{2}$ and the reference mappings $\mathcal {M}^{RA}$. Analogously, a mapping *m* such that $m \not \in \mathcal {M}^{S}$ and $m \in \mathcal {M}^{RA}$ will penalise standard recall, even though the aligned ontology $\mathcal {O}^{\mathcal {M}^{S}}$ can entail *m*. In this paper we adopt the notion of *semantic precision* and *recall* as defined in Eqs. 2 and 3 to mitigate the limitations of the standard measures (the interested reader please refer to [9, 10] for alternative definitions).

Semantic precision and recall, as presented in this paper, may still suffer from some limitations [11]. In order to reduce the impact of these limitations, when computing semantic precision and recall, equivalence mappings (≡) are split into two subsumption mappings ($\sqsubseteq $ and $\sqsupseteq $).

Note that when evaluating the mappings produced by a matching system against (incomplete) baseline mappings, only semantic recall should be taken into account.

2 $$ P_{(sem)} = \frac{\lvert\{m \in \mathcal{M}^{S} \mid \mathcal{O}^{\mathcal{M}^{RA}} \models m \}\rvert}{\lvert\mathcal{M}^{S}\rvert}~  $$

3 $$ R_{(sem)} = \frac{\lvert\{m \in \mathcal{M}^{RA} \mid \mathcal{O}^{\mathcal{M}^{S}} \models m \}\rvert}{\lvert\mathcal{M}^{RA}\rvert}  $$

An ontology is *incoherent* [12] if it contains logical errors in the form of unsatisfiable concepts. If the union of the input ontologies $\mathcal {O}_{1}$ and $\mathcal {O}_{2}$ and the reference mappings $\mathcal {M}^{RA}$ is incoherent, semantic precision and recall, as defined in Eqs. 2 and 3, may lead to unexpected results. In this case, mapping repair (e.g., [12–14]) techniques should be applied before computing semantic precision and recall.

## Methods

The Ontology Alignment Evaluation Initiative^7^ (OAEI) is an annual campaign for the systematic evaluation of ontology matching systems [1, 15–17]. The main objective is the comparison of ontology matching systems on the same basis and to enable the reproducibility of the results. The OAEI included 9 different tracks organised by different research groups and involving different matching tasks.

The novel *Disease and Phenotype* ^8^ track was one of the new additions in the OAEI 2016 campaign. The track aims at evaluating the performance of systems in a real-world use case where pairwise alignments between disease and phenotype ontologies are required.

**Fig. 1:**
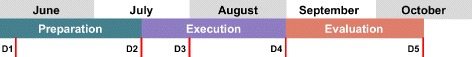
Phases of the OAEI 2016 *Disease and Phenotype* track. Important Dates: D1 (publication of preliminary datasets), D2 (final datasets released), D3 (system registration), D4 (system submission), D5 (publication of evaluation results and presentation in the Ontology Matching workshop [1])

The *Disease and Phenotype* track closely followed the OAEI phases as summarised in Fig. 1.

### Dataset

The *Disease and Phenotype* track comprises two matching tasks that involve the alignment of the Human Phenotype Ontology (HP), the Mammalian Phenotype Ontology (MP), the Human Disease Ontology (DOID), and the Orphanet Rare Disease Ontology (ORDO). Table 1 shows the metrics provided by BioPortal of these ontologies.

**Table 1:** Metrics of the track ontologies. Source: NCBI BioPortal on 2nd June 2016

Ontology	Number of axioms	Number of classes	Maximum depth	Avg. number of children
HP	137,289	11,786	15	3
MP	129,036	11,721	15	3
DOID	124,362	9248	12	3
ORDO	188,991	12,936	11	16

Note that the metric “average number of children” excludes the leaf nodes

**Task 1:** pairwise alignment of the HP and the MP ontologies (HP-MP matching task).

**Task 2:** pairwise alignment of the DOID and the ORDO ontologies (DOID-ORDO matching task).

### Preparation phase

As specified by the OAEI the ontologies and (public) reference alignments were made available in advance during the first week of June 2016. The ontologies and mappings were downloaded from BioPortal [18] on June 2nd.

The mappings were obtained using a script that, given a pair of ontologies, uses BioPortal’s REST API^9^ to retrieve all mappings between those ontologies. We focused only on *skos:closeMatch* (BioPortal) mappings^10^ as suggested in [19], and we represented them as equivalence mappings.^11^ The BioPortal-based alignment between HP and MP consisted in 639 equivalence mappings, while the alignment between DOID and ORDO included 1,018 mappings. Mappings were made available in both RDF Alignment and OWL 2 formats.

The preparatory phase gives the opportunity to both OAEI track organisers and participants to find and correct problems in the datasets. During this phase we noticed that the BioPortal mappings were highly incomplete.^12^ Hence, the participants were notified that the BioPortal-based mappings were to be used as a *baseline* and not as a *gold standard* reference alignment. Given the limitations of the BioPortal mappings we were in need of creating a (blind) *consensus reference alignment* to perform the (automatic) evaluation (see details in the Evaluation phase section).

All (open) OAEI datasets were released on July 15th, 2016 and did not evolve after that.

### Execution phase

System developers had to implement a simple interface and to wrap their tools including all required libraries and resources in order to use the SEALS infrastructure.^13^ The use of the SEALS infrastructure ensures that developers can perform a full evaluation locally and eases the reproducibility and comparability of the results.

This phase was conducted between July 15th and August 31st, 2016. During this time OAEI organisers attended technical issues reported by the developers. We also requested system developers to register their systems and their intention to participate in the different OAEI tracks by July 31st. Thirty systems were registered, from which 14 seemed potential participants of the *Disease and Phenotype* track.

### Evaluation phase

Participants were required to submit their wrapped tools by August 31st, 2016. From the 30 registered systems only 21 were finally submitted, and 13 were annotated (by the system developers) as participants of the *Disease and Phenotype* track. The final results were published on the OAEI website by October 15th. 

The evaluation for the *Disease and Phenotype* track was semi-automatic with support of the SEALS infrastructure. Systems were evaluated according to the following criteria for each of the matching tasks of the *Disease and Phenotype* track:

- Semantic precision and recall with respect to the *consensus alignments*.
- Semantic recall with respect to manually generated mappings.
- Manual assessment of unique mappings produced by a participant system.

Algorithm 1 formalizes the steps followed in the evaluation for each of the *Disease and Phenotype* matching tasks. The following subsections below comment on the main points of the evaluation process.

**Consensus alignments.** The *consensus alignments* are automatically generated based on the alignments produced by the participating systems in each of the matching tasks of the track. For the evaluation we have selected the *consensus alignments* of vote =2 (i.e., mappings suggested by two or more systems) and vote =3 (i.e., mappings suggested by three or more systems). In the case where both an equivalence and a subsumption mapping contribute to the consensus, the equivalence relationship prevails over the subsumption. The use of vote =2 and vote =3 was motivated by our experience in the creation of consensus alignments [20]. Consensus alignments with vote ≥4 are typically highly precise but also very incomplete unless the number of contributing systems is significant.^14^ Note that, when there are several systems of the same family (i.e., systems participating with several variants), their (voted) mappings are only counted once in order to reduce bias.^15^

Note that consensus alignments have numerous limitations. It allows us to compare how the participating systems perform only in relation to each other. Some of the mappings in the consensus alignments may be erroneous (false positives), as it only requires 2 or 3 systems to agree on the erroneous mappings they find. Furthermore, the consensus alignments may not be complete, as there will likely be correct mappings that no or only one system is able to find. Nevertheless, consensus alignments help to provide some insights into the performance of a matching system.

**Semantic precision and recall.** As introduced in the Preliminaries section, the semantic precision and recall take into account the implicit knowledge derived from the ontologies and the mappings via OWL 2 reasoning.^16^ Hence, the methods *SemanticPrecision* and *SemanticRecall* in Algorithm 1 receive as input a set of mappings $\mathcal {M}$ and a coherent ontology $\mathcal {O}$. Both methods return as output the value of $\frac {|\mathcal {M}'|}{|\mathcal {M}|}$ where $\mathcal {M}'$ is a subset of $\mathcal {M}$ such that the mappings $m \in \mathcal {M}'$ are entailed by $\mathcal {O}$ (i.e., $\mathcal {O} \models m$).

**Manually generated mappings.** These reference mappings were created through manual curation by eight disease informatics experts, who are authors of this paper, all working within or for the pharmaceutical industry for three areas of phenotype and disease; namely carbohydrate and glucose metabolism, obesity and breast cancer. These sets of reference mappings comprised of 29 pairwise mappings between HP and MP and 60 pairwise mappings between DOID and ORDO across the three areas. They included some relationships of equivalence, but most of them represented subsumption relationships. The three areas were selected as representative samples which were known already to be present across the four source ontologies. Inclusion of these manually defined mappings enabled a real-world evaluation of recall for the two matching tasks. The future editions of the track will increase the number of manual mappings through inclusion of additional areas relevant to the phenotype and disease domain.

**Unique mappings and manual assessment.** Unique mappings are mappings generated by an ontology matching system that have not been (explicitly) suggested by any of the other participating systems, nor entailed by the aligned ontology using the consensus alignment with vote =2 $\left ({\mathcal {O}^{\mathcal {M}^{R\!A}_{c2}}}\right)$. The method *UniqueMappings* in Algorithm 1 receives as input a set of mappings $\mathcal {M}$ and the (coherent) ontology ${\mathcal {O}^{\mathcal {M}^{R\!A}_{c2}}}$ and returns as output $\mathcal {M}'$ where $\mathcal {M}'\subseteq \mathcal {M}$ such that the mappings $m \in \mathcal {M}'$ are not entailed by ${\mathcal {O}^{\mathcal {M}^{R\!A}_{c2}}} \left (i.e., {\mathcal {O}^{\mathcal {M}^{R\!A}_{c2}}} \not \models m\right)$.

Manual assessment over unique mappings has been performed by an expert in disease informatics from the pharmaceutical industry. This assessment aims at complementing the evaluation against the consensus alignments of those mappings that, although being suggested or voted by only one matching system, may still be correct. We have focused the assessment on unique “equivalence” mappings and we have manually evaluated up to 30 mappings for each system in order to (roughly) estimate the percentage of correct mappings (i.e., precision, *P*_*m*_ in Algorithm 1) and the positive/negative contribution to the total number of unique mappings (*PC* and *NC* in Algorithm 1), that is, the weight of the correct (i.e., true positives) and incorrect (i.e., false positives) mappings. Intuitively, the positive contribution (see Eq. 4) of a system producing a small set of unique mappings will most likely be smaller than a system producing a larger set of unique (and mostly correct) mappings. The negative contribution (see Eq. 5) will weight the number of incorrect unique mappings with respect to the total. Negative and positive contributions, for a set of unique mappings $\mathcal {U}^{S}_{i}$ computed by a system *i*, are defined as follows:

4 $$ {}{\mathsf{PositiveContribution}}\left(\mathcal{U}^{S}_{i}\right) = \frac{\big\lvert\mathcal{U}^{S}_{i}\big\rvert \cdot {\mathsf{Precision}}\left(\mathcal{U}^{S}_{i}\right)}{\sum_{j=1}^{n}\big\lvert\mathcal{U}^{S}_{j}\big\rvert}~  $$

5 $$ {}{\mathsf{NegativeContribution}}\left(\mathcal{U}^{S}_{i}\right) = \frac{\big\lvert\mathcal{U}^{S}_{i}\big\rvert \cdot \left(1-\mathsf{Precision}\left(\mathcal{U}^{S}_{i}\right)\right)}{\sum_{j=1}^{n}\big\lvert\mathcal{U}^{S}_{j}\big\rvert}~  $$

## Results

We have run the evaluation of the *Disease and Phenotype* track in a Ubuntu Laptop with an Intel Core i7-4600U CPU @ 2.10 GHz x 4 and allocating 15 Gb of RAM. From the 13 systems registered to the track (out of 21 OAEI participants), 11 systems have been able to cope with at least one of the *Disease and Phenotype* matching tasks within a 24 h time frame. Results for all OAEI tracks have been reported in [1].

### Participating systems

**AML** [21, 22] is an ontology matching system originally developed to tackle the challenges of matching biomedical ontologies. While its scope has since expanded, biomedical ontologies have remained one of the main drives behind its continued development. AML relies on the use of background knowledge and it also includes mapping repair capabilities.

**DiSMatch** [23] estimates the similarity among concepts through textual semantic relatedness. DiSMatch relies on a biomedical domain-adapted variant of a state-of-the-art semantic relatedness measure [24], which is based on Explicit Semantic Analysis.

**FCA-Map** [25] is an ontology matching system based on Formal Concept Analysis (FCA). FCA-Map attempts to push the envelope of the FCA to cluster the commonalities among classes at various levels.

**LogMap** [26, 27] relies on lexical and structural indexes to enhance scalability. It also incorporates approximate reasoning and repair techniques to minimise the number of logical errors in the aligned ontology.

**LogMapBio** [28] extends LogMap to use BioPortal [18] as a (dynamic) provider of mediating ontologies, instead of relying on a few preselected ontologies. LogMapBio retrieves the most suitable top-10 ontologies for the matching task.

**LogMapLt** is a “lightweight” variant of LogMap, which essentially only applies (efficient) string matching techniques.

**LYAM++** [29] is a fully automatic ontology matching system based on the use of external sources. LYAM++ applies a novel orchestration of the components of the matching workflow [30].

**PhenomeNET** [31] alignment system comes in three flavours, which rely on three different versions of the PhenomeNET ontology [32]. PhenomeNET-Plain (PhenoMP) relies on a plain ontology which only uses the axioms provided by the HP ontology and the MP ontology. PhenomeNET-Map (PhenoMM) utilizes additional lexical equivalence axioms between HP and MP provided by BioPortal. Finally, PhenomeNET-Full (PhenoMF) relies on an extended version of the PhenomeNET ontology with equivalence mappings to the DOID and ORDO ontologies obtained via BioPortal and the AML matching system [21].

**XMap** [33] is a scalable matcher that implements parallel processing techniques to enable the composition of basic ontology matchers. It also relies on the use of external resources such as the UMLS Metathesarus [34].

### Use of specialised background knowledge

The use of (specialised) background knowledge is allowed in the OAEI, but participants are required to specify which sources their systems rely on to enhance the matching process. AML has three sources of background knowledge which can be used as mediators between the input ontologies: the Uber Anatomy Ontology [35] (Uberon), the Human Disease Ontology [4] (DOID) and the Medical Subject Headings^17^ (MeSH). LYAM++ also makes use of the Uberon ontology [35]. LogMapBio uses BioPortal [18] as dynamic mediating ontology provider, while LogMap uses normalisations and spelling variants from the general (biomedical) purpose UMLS Lexicon.^18^ XMAP uses synonyms provided by the UMLS Metathesaurus [34]. Finally, PhenoMM, PhenoMF and PhenoMP rely on different versions of the PhenomeNET^19^ ontology [32] with variable complexity as described above.

### Evaluation against BioPortal (baseline) mappings

Table 2 shows the results in terms of semantic recall against the baseline mappings extracted from BioPortal as described in the “Methods” Section (Preparation phase). In the DOID-ORDO task, LYAM++ failed to complete the task while PhenoMM and PhenoMP produced empty mapping sets.

**Table 2:** Recall against BioPortal (baseline) mappings

System	AML	DiSMatch	FCA-Map	LYAM++	LogMap	LogMapBio	LogMapLt	PhenoMF	PhenoMM	PhenoMP	XMap
HP-MP	1.0	0.25	0.998	0.014	0.997	1.0	0.994	1.0	1.0	0.412	0.995
DOID-ORDO	0.993	0.048	0.984	-	0.942	0.950	0.943	0.994	0.0	0.0	0.967

BioPortal mappings mostly represent correspondences with a high degree of lexical similarity and, as expected, most of the systems managed to produce alignments with a very high recall. DiSMatch, LYAM++, PhenoMM (in the DOID-ORDO task) and PhenoMP were the exception and produced very low results with respect to the baseline mappings. As mentioned in the “Methods” Section, since the BioPortal mappings were highly incomplete, the results in terms of (semantic) precision were not significant. For this reason, we needed to create consensus alignments for each task.

### Creation of consensus alignments

In the MP-HP matching task 11 systems were able to produce mappings. Mappings voted by LogMap and PhenomeNET families were only counted once, and hence there were 7 independent system groups contributing to the consensus alignment. In the DOID-ORDO matching task 8 systems generated mappings and there were 6 independent system groups contributing to the consensus alignment.

Table 3 (resp. Table 4) shows the size of the different consensus alignments from vote =1, i.e., mappings suggested by one or more system groups, to vote =7 (resp. vote =6), i.e., mappings suggested by all system groups, in the HP-MP matching task (resp. DOID-ORDO task). It is noticeable that in the HP-MP task there were 0 mappings where all systems agreed, while in the DOID-ORDO task there were only 36. The number of mappings suggested by one system or more is specially large because PhenomeNET systems produce a large number of subsumption mappings. If only equivalence mappings of PhenomeNET systems are taken into account, the number of mappings with vote =1 would be 3433 in the HP-MP task and 2708 in the DOID-ORDO task.

**Table 3:** Consensus alignments for the HP-MP matching task

Min. Votes	1	2	3	4	5	6	7
Mappings	217039	2308	1588	1287	677	152	0

Seven (family) system groups contributing

**Table 4:** Consensus alignments for the DOID-ORDO matching task

Min. Votes	1	2	3	4	5	6
Mappings	50,998	1883	1617	1447	991	36

Six (family) system groups contributing

As described in the “Methods” Section we have selected the consensus alignments of vote =2 and vote =3. These consensus alignments for HP-MP contain 2308 and 1588 mappings, respectively; while for DOID-ORDO they include 1883 and 1617 mappings, respectively. Table 5 shows some examples of mappings included with the consensus alignments of vote =2 and vote =3. Also shown are some examples of manually created mappings and (correct/incorrect) unique mappings from ontology matching systems.

**Table 5:** Example mappings in the *Disease and Phenotype* track

Entity 1	Entity 2	Rel.	Source
x-linked chondrodysplasia punctata (DOID_0060292)	Chondrodysplasia punctata (Orphanet_93442)	≡	(only) consensus alignment vote=2
Meningeal melanomatosis (DOID_8243)	Diffuse leptomeningeal melanocytosis	≡	Consensus alignment vote=3
	(Orphanet_252031)		
Reactive arthritis (DOID_6196)	Reactive arthritis (Orphanet_29207)	≡	Consensus alignment vote=3
Hypoplastic scapulae (HP_0000882)	Short scapula (MP_0004340)	≡	(only) consensus alignment vote=2
Macrocytic anemia (HP_0001972)	Macrocytic anemia (MP_0002811)	≡	Consensus alignment vote=3
Unerupted tooth (HP_0000706)	Failure of tooth eruption (MP_0000121)	≡	Consensus alignment vote=3
Breast leiomyosarcoma (DOID_5285)	Rare malignant breast tumor	$\sqsubseteq $	Manually created
	(Orphanet_180257)		
Abnormality of body weight (HP_0004323)	Abnormal body weight (MP_0001259)	≡	Manually created
Microcephaly (HP_0000252)	Decreased brain size (MP_0000774)	≡	AML unique mapping (correct)
Skeletal dysplasia (HP_0002652)	Abnormal skeletal muscle morphology	≡	AML unique mapping (incorrect)
	(MP_0000759)		
Carbohydrate metabolism disease (DOID_0050013)	Disorder of carbohydrate metabolism	≡	LogMapBio unique mapping (correct)
	(Orphanet_79161)		
Spinocerebellar ataxia type 35 (DOID_0050982)	Transglutaminase 6 (Orphanet_279644)	≡	LogMapBio unique mapping (incorrect)
Female hypogonadism (HP_0000134)	Small ovary (MP_0001127)	≡	PhenoMF unique mapping (correct)

### Results against consensus alignments

The union of the input ontologies together with the consensus alignments or the mappings computed by each of the systems was coherent and thus, we did not require to repair any of the mapping sets to calculate the semantic precision and recall. Note that the downloaded ontology versions from BioPortal did not contain any explicit or implicit disjointness. Tables 6 and 7 show the results achieved by each of the participating systems against the consensus alignments with vote =2 and vote =3. In the DOID-ORDO task, LYAM++, PhenoMM and PhenoMP failed to produce mappings and they were not included in Table 7.

**Table 6:** Results against consensus alignments with vote =2 and vote =3 in the HP-MP task

System-mappings	Mappings	Precision-2	F-Measure-2	Recall-2	Precision-3	F-Measure-3	Recall-3
*BioPortal (baseline)*	*639*	*1.00*	*0.50*	*0.33*	*1.00*	*0.60*	*0.43*
*AML*	1755	0.93	0.86	0.80	0.85	0.90	0.94
*DiSMatch*	644	0.55	0.30	0.21	0.45	0.28	0.20
*FCA* − *Map*	1590	0.98	0.85	0.75	0.94	0.93	0.92
*LYAM* + +	381	0.41	0.12	0.07	0.17	0.06	0.04
*LogMap*	2011	0.94	0.92	0.91	0.77	0.86	0.97
*LogMapBio*	2151	0.92	0.92	0.93	0.75	0.85	0.98
*LogMapLt*	667	1.00	0.51	0.34	1.00	0.62	0.45
*PhenoMF*	204,089	0.76	0.83	0.92	0.63	0.76	0.95
*PhenoMM*	198,149	0.77	0.83	0.91	0.64	0.76	0.94
*PhenoMP*	169,660	0.78	0.67	0.58	0.64	0.57	0.51
*XMap*	650	1.00	0.50	0.33	1.00	0.61	0.44

Precision and Recall represent their semantic variants

**Table 7:** Results against consensus alignments with vote =2 and vote =3 in the DOID-ORDO task

System-mappings	Mappings	Precision-2	F-Measure-2	Recall-2	Precision-3	F-Measure-3	Recall-3
*BioPortal (baseline)*	*1018*	*0.99*	*0.71*	*0.55*	*0.99*	*0.76*	*0.62*
*AML*	2098	0.85	0.91	0.97	0.78	0.87	1.00
*DiSMatch*	335	0.23	0.08	0.05	0.19	0.07	0.04
*FCA* − *Map*	1803	0.97	0.96	0.96	0.89	0.94	0.99
*LogMap*	1667	0.95	0.91	0.88	0.91	0.92	0.94
*LogMapBio*	1804	0.92	0.91	0.90	0.86	0.90	0.95
*LogMapLt*	1000	0.99	0.72	0.56	0.99	0.76	0.62
*PhenoMF*	40,612	0.95	0.89	0.83	0.95	0.94	0.92
*XMap*	1030	0.98	0.72	0.57	0.98	0.77	0.63

Precision and Recall represent their semantic variants

We deliberately did not rank the systems since, as mentioned in the “Methods” section, the consensus alignments may be incorrect or incomplete. We have simply highlighted the systems producing results relatively close to the consensus alignments. For example, in the HP-MP task, LogMap is the system producing an alignment that is closer to the mappings voted by at least 2 systems, while FCA-MAP produces results very close to the consensus alignments with vote =3.

The use of semantic precision and recall allowed us to provide a fair comparison for the systems PhenoMF, PhenoMM and PhenoMP. These systems discover a large set of subsumption mappings that are not explicit in the reference alignments, but they are still valid (i.e., they are entailed by the aligned ontology using the reference alignment). For example, the standard precision of PhenoMF in the HP-MP task is 0.01 while the semantic precision reaches the value of 0.76.

Tables 6 and 7 also include the results of BioPortal mappings against the consensus alignments. Precision values are perfect, but recall is very low, which confirms our intuitions (recall “Preparation phase” section) about the incompleteness of BioPortal mappings.

It is striking how XMap and LogMapLt produced results very similar to the ones obtained by the BioPortal mappings. Closer scrutiny of these results showed us that the computed mappings were indeed very similar to the BioPortal mappings (i.e., the F-measure of XMap and LogMapLt against the *baseline* mappings provided by BioPortal is ≥ 0.95 in both tasks).

This could be expected for LogMapLt, since it only relies on simple string matching techniques as the matching system underlying BioPortal [36]. However, the results for XMap are unexpected since it produced top-results in the other biomedical-themed tracks of the OAEI 2016 [1].

### Results against manually created mappings

Table 8 shows the results in terms of semantic recall against the manually created alignments. The results obtained in the HP-MP are relatively large positive values in general, especially for PhenoMF and PhenoMM that achieve a semantic recall of 0.90. The numbers for the DOID-ORDO, however, are much smaller values and only LogMap, LogMapBio and DisMatch are able to discover a few of the manually curated mappings. LogMapBio obtained the best semantic recall value with 0.17, which is far from the top results in the HP-MP task. The aforementioned results are also reflected when considering the consensus alignments. In the HP-MP task, both the consensus alignments with vote 2 and 3 obtained reasonably good results. However the picture changes dramatically in the DOID-ORDO task where none of the manually curated mappings are covered by the mappings agreed by 2 or more systems. The most likely explanation for this result is that the manual mappings for DOID-ORDO represent more complex subsumption mappings which were not possible to (semantically) derive for the other mappings. Table 8 also shows the results for the BioPortal mappings, which, as expected, have a coverage of curated mappings very similar to the obtained by LogMapLt and XMap systems.

**Table 8:** Results against curated alignments

System-mappings	HP-MP task		DOID-ORDO task	
	Standard recall	Semantic recall	Standard recall	Semantic recall
*BioPortal (baseline)*	*0.17*	*0.52*	*0.00*	*0.00*
*AML*	0.28	0.76	0.00	0.00
*DiSMatch*	0.07	0.14	0.02	0.03
*FCA* − *Map*	0.21	0.62	0.00	0.00
*LYAM* + +	0.00	0.00	-	-
*LogMap*	0.24	0.66	0.02	0.12
*LogMapBio*	0.28	0.69	0.03	0.17
*LogMapLt*	0.17	0.52	0.00	0.00
*PhenoMF*	0.90	0.90	0.00	0.00
*PhenoMM*	0.90	0.90	-	-
*PhenoMP*	0.83	0.83	-	-
*XMap*	0.17	0.52	0.00	0.00
*Consensus* *vote* = 1	0.90	0.90	0.05	0.20
*Consensus* *vote* = 2	0.31	0.79	0.00	0.00
*Consensusvote* = 3	0.24	0.66	0.00	0.00

The use of semantic recall together with the standard measure, as in previous section, allowed us to provide more realistic results and a fair comparison with the PhenomeNET family systems. As it can be observed in the HP-MP task (Table 8), the standard recall, unlike the semantic recall, obtained by the other participants was very low and not comparable to the PhenomeNET family systems.

While the top performing algorithms were able to detect equivalence matches across whole source ontologies for the two mapping tasks giving high F-measures (Tables 6 and 7), it is clear from detection of the curated alignments that these proved much more difficult with a trend for lower semantic recall across both tasks (Table 8). This result was not surprising because the curated alignments mostly comprised of subsumption relationships rather than equivalence. Table 5 shows two examples of curated mappings; the equivalence mapping between *abnormality of body weight* and *abnormal body weight* was suggested by at least one the systems, while the subsumption mapping between *breast leiomyosarcoma* and *rare malignant breast tumor* was not discovered by any of the systems.

### Results for manual assessment of unique mappings

Tables 9 and 10 show the results of the manual assessment of the unique mappings generated by the participating systems. As mentioned in the “Methods” section we manually analysed up to 30 unique equivalence mappings for each system to estimate the precision of the generated mappings not agreed with other systems. Table 5 shows examples of unique mappings computed by AML, LogMapBio and PhenoMF. Note that, we focus on equivalence mappings since PhenomeNET systems produce a large amount of (unique) subsumption mappings.

**Table 9:** Manual assessment of unique mappings and estimated positive and negative contribution in the HP-MP task

System-mappings	Unique mappings	Precision	Positive contrib.	Negative contrib.
*BioPortal (baseline)*	*0*	*-*		
*AML*	122	0.87	8.63%	1.33%
*DiSMatch*	291	0.83	19.80%	3.96%
*FCA* − *Map*	26	0.96	2.04%	0.08%
*LYAM* + +	226	0.70	12.91%	5.53%
*LogMap*	130	0.93	9.90%	0.71%
*LogMapBio*	176	0.93	13.40%	0.96%
*LogMapLt*	0	-	-	-
*PhenoMF*	89	1.00	7.27%	0.00%
*PhenoMM*	85	1.00	6.94%	0.00%
*PhenoMP*	80	1.00	6.53%	0.00%
*XMap*	0	-	-	-
*Total*	*1225*	*0.91*	*87.42%*	*12.58%*

**Table 10:** Manual assessment of unique mappings and estimated positive and negative contribution in the DOID-ORDO task

System-mappings	Unique mappings	Precision	Positive contrib.	Negative contrib.
*BioPortal (baseline)*	*5*	*0.40*		
*AML*	308	0.87	30.40%	4.68%
*DiSMatch*	259	0.40	11.80%	17.70%
*FCA* − *Map*	61	0.83	5.79%	1.16%
*LogMap*	80	0.90	8.20%	0.91%
*LogMapBio*	144	0.97	15.85%	0.55%
*LogMapLt*	7	0.50	0.40%	0.40%
*PhenoMF*	3	1.00	0.34%	0.00%
*XMap*	16	0.56	1.03%	0.80%
*Total*	*878*	*0.75*	*73.81%*	*26.19%*

BioPortal mappings, as expected, contains a very low number of unique mappings in the DOID-ORDO task and no unique mappings in the HP-MP task.

It is noticeable in the HP-MP task that, although DiSMatch and LYAM++ produced very low results with respect to the consensus alignments (see Table 3), the positive contribution of their unique mappings is one of the highest. Nevertheless, their negative contribution has also an important weight. PhenomeNET systems produced the most precise set of unique mappings although their positive contribution was lower than other systems.

In the DOID-ORDO matching task, AML’s unique mappings contains the higher number of true positives with a reasonable number of false positives. LogMapBio provided the best trade-off between positive and negative contribution.

The last row in Tables 9 and 10 shows (excluding BioPortal mappings) the total number of unique mappings, its (average) precision, and the total (aggregated) positive and negative contribution.

### Results in the OAEI interactive matching track

The OAEI interactive track^20^ aims at offering a systematic and automated evaluation of matching systems with user interaction to compare the quality of interactive matching approaches in terms of F-measure and number of required interactions. The interactive track relies on the datasets of the OAEI tracks: *Conference*, *Anatomy*, *Largebio*, and *Disease and Phenotype*; and it uses the reference alignments of each track as *oracle* in order to simulate the interaction with a domain expert with variable error rate [1].

In this section we briefly present the results with the *Disease and Phenotype* datasets in the OAEI 2016 interactive track, which represents a side contribution of the work presented in this paper. For more details and results, the interested reader please refer to state-of-the-art papers on *interactive ontology alignment* [1, 37–39].

The consensus alignment with vote =3 was used as oracle in the *Disease and Phenotype* interactive track. Table 11 shows the obtained F-measure by AML and LogMap when simulating an interaction with a perfect user (i.e., always gives the correct answer when asked about the validity of a mapping).^21^ Both systems increase the F-measure with respect to the non-interactive results (see Tables 6 and 7) with a gain between 0.03 and 0.11. It is noticeable that the number of required requests by LogMap is around 4-5 times larger than AML.

**Table 11:** Results in the OAEI interactive track

Task	System	F-measure	Gain	Requests
HP-MP	*AML*	0.93	0.03	388
	*LogMap*	0.97	0.11	1928
DOID-ORDO	*AML*	0.96	0.09	413
	*LogMap*	0.99	0.07	1602

## Discussion

The OAEI has been proven to be an effective campaign to improve ontology matching systems. As a result, available techniques are more mature and robust. Nevertheless, despite the impressive state-of-the-art technology in ontology alignment, new matching tasks like those presented in this paper are very important for the OAEI campaign since they introduce new challenges to ontology alignment systems. For example, our preliminary tests with the *Disease and Phenotype* dataset revealed that only the 2015 versions of AML and LogMap, among the systems participating in the OAEI 2015, were able to cope with the track ontologies.

In the OAEI 2016 campaign there were 11 systems that were able to produce results in at least one of the *Disease and Phenotype* matching tasks. The four systems: AML, FCA-Map, LogMap (and its *Bio* variant) and PhenoMF produced alignments relatively close to the consensus alignments for the *Disease and Phenotype* evaluation tasks as described in this paper. The results against curated alignments proved to be more challenging since they go beyond equivalent matches to include matches of semantic similarity, especially subsumption relationships. This finding suggests that while the systems performed well enough for detection of equivalent mappings, in future it would be good to improve their performance for detection of semantic similarity matches. For example, PhenomeNET systems showed potential advantage though exploiting a specialised background knowledge embedded within the system. LYAM++ is also specialised in the use of background knowledge, but it did not perform well in the *Disease and Phenotype* track, unlike in the OAEI Anatomy track, probably due to the lack of a suitable source of background knowledge for this track.

The OAEI also includes two biomedical-themed tracks, namely *Anatomy* and *Largebio* [1]. The complexity of the matching tasks is similar to the Anatomy track in terms of ontology size and expressiveness, while the Largebio tasks represent a significant leap in complexity with respect to the other OAEI test cases. The main differences with respect to the evaluation in the *Disease and Phenotype* track are the following: *(i)* we constructed two consensus reference alignments, unlike the Anatomy track where there exist a curated reference alignment [40] and the Largebio track where the reference alignment has been extracted from the UMLS Metathesaurus [8]; *(ii)* we performed an evaluation with respect to manually created mappings and a manual assessment of unique mappings produced by participating systems; and *(iii)* we used semantic precision and recall together with the standard measures.

The findings of the *Disease and Phenotype* evaluation show the potential of the top performing ontology matching systems that could help to automate the workflow of curators, who maintain ontology mapping services in numerous domains such as the disease and phenotype domain. Furthermore, the constructed consensus alignments substantially improve available mapping sets provided by BioPortal.

## Conclusions

We have presented the methodology followed in the novel *Disease and Phenotype* track and the results in the OAEI 2016. The top systems in the track coped well with the detection of equivalence matches, but struggled to detect subsumption matches. This deserves more attention in the future development of ontology matching systems.

The Pistoia Alliance Ontologies Mapping project has gained much value from participation in the 2016 OAEI campaign through sponsorship and design of this new track on *Disease and Phenotype*. We believe that there is a real need for ontology matching algorithm developers to collaborate with ontology curators to improve the scale and quality of workflows necessary to build and maintain ontology mapping resources.

We are in an exploding information age with increasing amounts of human biology and genetics data in particular from sequencing technology improvements, biobanks and smart portable devices. This drives the need for stronger ontological standards, tools and services for ontology mapping to enable more efficient application of all this information. We expect that the *Disease and Phenotype* track will evolve in future campaigns as a strong use case which is widely applicable in the life sciences and beyond.

### Evolution of the track

The OAEI 2017 will include a new edition of the track, which will be composed by the same tasks as in 2016 (with updated ontology versions) and two additional tasks requiring the pairwise alignment of:

- HP and MESH (Medical Subject Headings) ontologies; and
- HP and OMIM (Online Mendelian Inheritance in Man) ontologies.

The alignment between HP and MESH is a new requirement of the Pistoia Alliance Ontologies Mapping project, while the mapping between HP and OMIM is placed within the scope of the Research Council of Norway project *BigMed* to improve the suggested genes associated to a given phenotype in state of the art tools like *PhenoTips* [41].

In the future editions of the *Disease and Phenotype* track, apart from including new datasets and updated versions, we aim to enhance the evaluation in a number of ways. We will consider *new* *metrics* like the mapping incoherence [12], the functional coherence [42] or the redundancy (minimality) [43] to evaluate the computed alignments. We also intend to redefine the *notion* *of* *semantic* *precision* *and* *recall*, using the using the semantic closure of the (aligned) ontologies, in order to include the cases where the aligned ontology is incoherence (i.e., contains unsatisfiable classes).

We plan to increase the number of *manually* *generated* *mappings* considering additional areas relevant to the phenotype and disease domain. In addition, we will also work towards the semi-automatic creation of *gold* *standard* reference alignments for the tasks by combining the consensus alignments and the manually generated mappings.

## Endnotes

^1^ http://www.pistoiaalliance.org/projects/ontologies-mapping

^2^ https://pistoiaalliance.atlassian.net/wiki/display/PUB/Ontologies+Mapping+Resources

^3^ The contents of this paper have been partially reported in the OAEI 2016 annual report [1], published within the “informal” proceedings of the Ontology Matching workshop [44].

^4^ http://www.orphadata.org/cgi-bin/inc/ordo_orphanet.inc.php

^5^ https://www.w3.org/TR/owl2-overview/

^6^ Typically automatic, although there are systems that also allow human interaction

^7^ http://oaei.ontologymatching.org/

^8^ http://oaei.ontologymatching.org/2016/phenotype/

^9^ http://data.bioontology.org/documentation%23Mapping

^10^ https://www.bioontology.org/wiki/index.php/BioPortal_Mappings

^11^ We did not consider mappings labelled as *skos:exactMatch* since they represent correspondences between entities with the same URI, and thus these mappings are redundant if translated into OWL 2 axioms.

^12^ Our tests with last year participants revealed a large amount of missing valid mappings. The “Results” section quantifies this degree of incompleteness.

^13^ http://oaei.ontologymatching.org/2016/seals-eval.html

^14^ We may consider vote ≥ 4 in future editions of the *Disease and Phenotype* track as the contributing participants increase.

^15^ There could still be some bias through systems exploiting the same resource, e.g., UMLS.

^16^ We rely on the OWL 2 reasoner HermiT [45].

^17^ http://bioportal.bioontology.org/ontologies/MESH

^18^ https://www.nlm.nih.gov/pubs/factsheets/umlslex.html

^19^ http://aber-owl.net/ontology/PhenomeNET

^20^ http://oaei.ontologymatching.org/2016/interactive/

^21^ From the *Disease and Phenotype* track participating systems only AML, LogMap and XMap implement an interactive algorithm. We have discarded XMap from the results since its number of oracle/user requests was very low in the *Disease and Phenotype* track.

## Abbreviations

DL: Description logics
DOID: Disease ontology
F: F-measure
HP: Human phenotype ontology
M: Mappings
MP: Mammalian phenotype ontology
O: Ontology
OA: Ontology alignment
OAEI: Ontology alignment evaluation initiative
ORDO: Orphanet rare disease ontology
OWL: Web ontology language
P: Precision
R: Recall
RA: Reference alignment
RDF: Resource description framework
